# Supplementary figures and images for: Effect of a blood culture collection bundle on decreasing the contamination rate
Source: PLoS One. 2024 Dec 31;19(12):e0314649. doi: 10.1371/journal.pone.0314649 (PMC11687820; doi:10.1371/journal.pone.0314649)

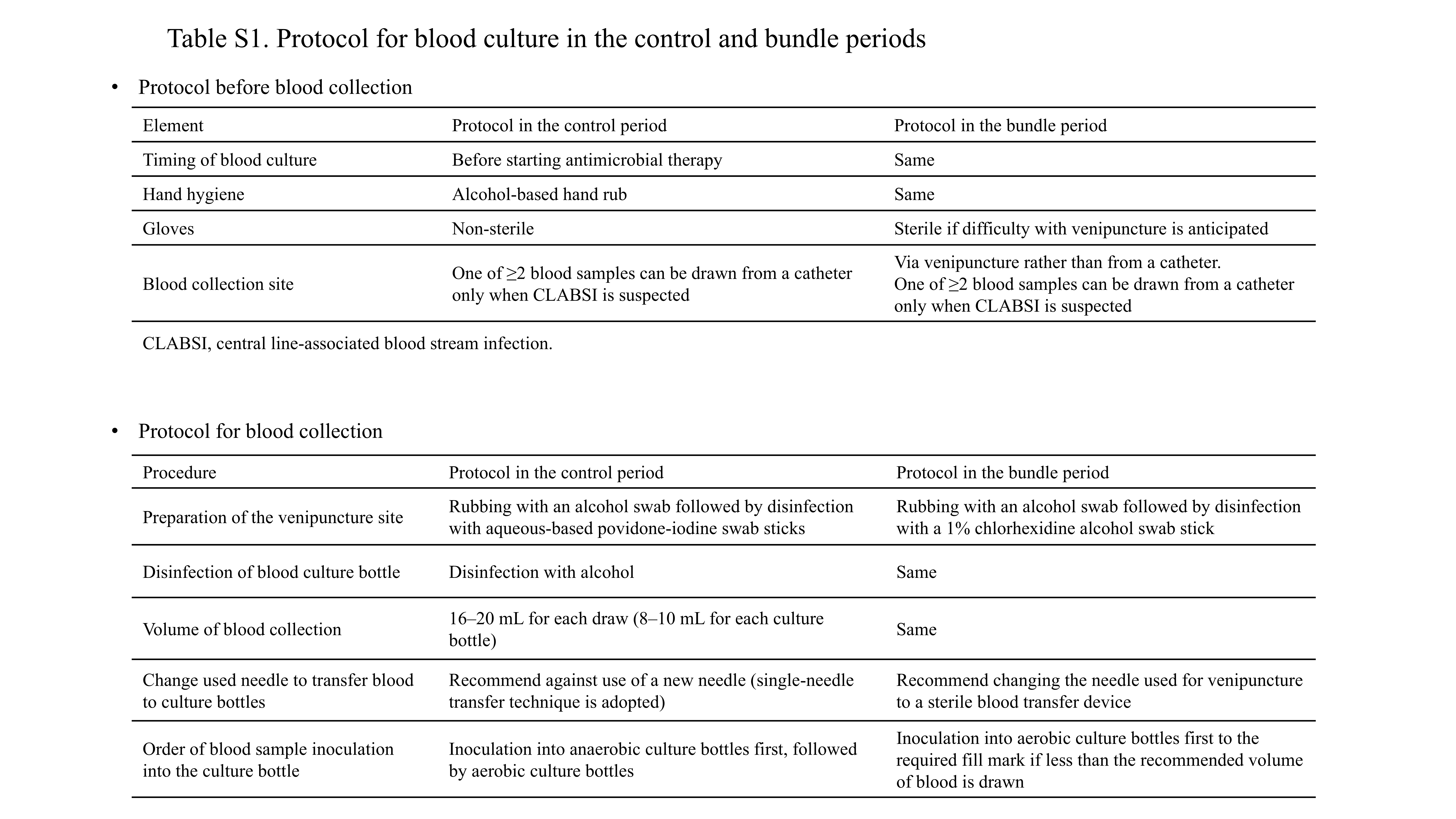

Supplement: S1 Table — (TIF) [file pone.0314649.s001.TIF]

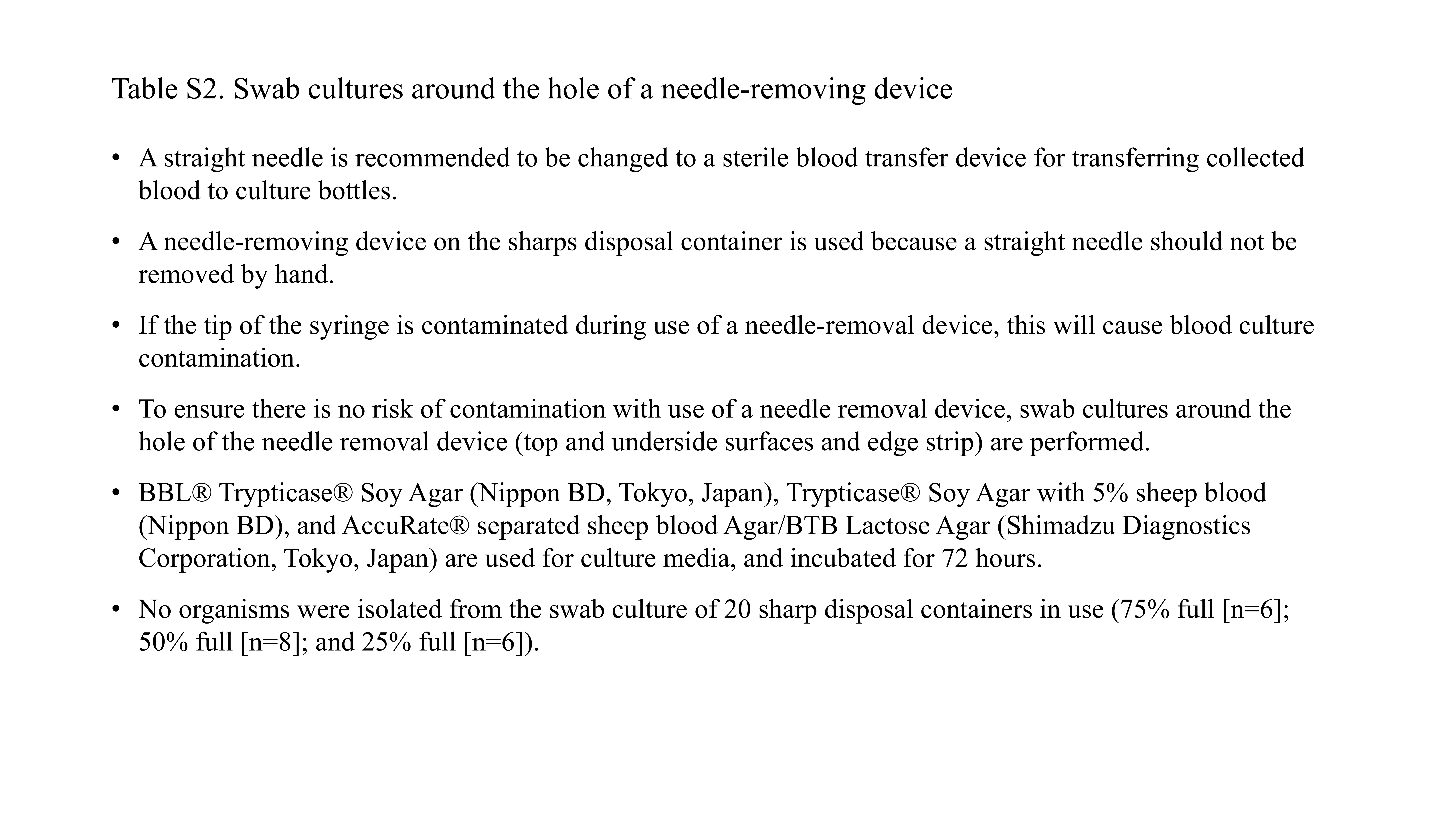

Supplement: S2 Table — (TIF) [file pone.0314649.s002.TIF]

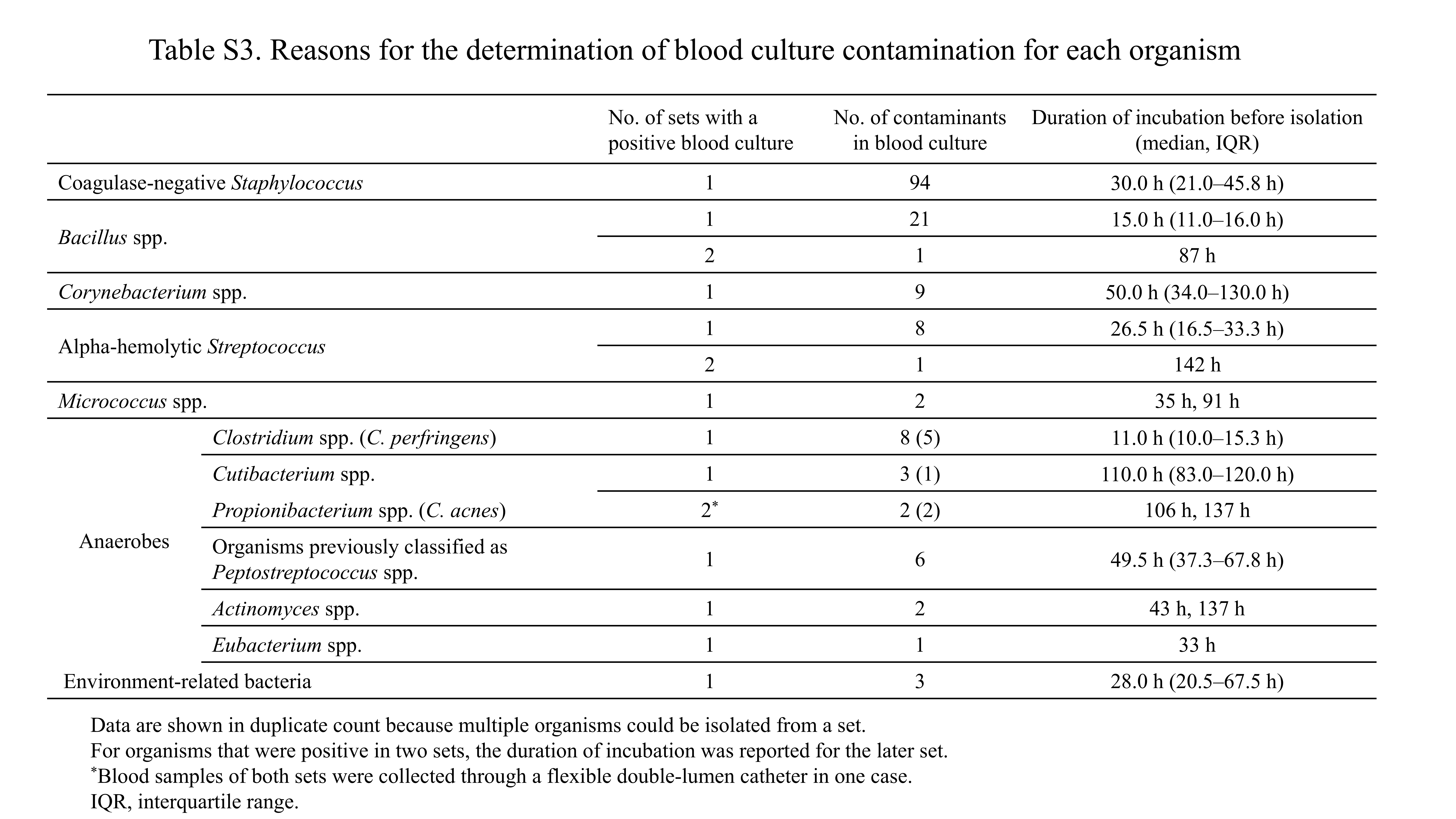

Supplement: S3 Table — (TIF) [file pone.0314649.s003.TIF]

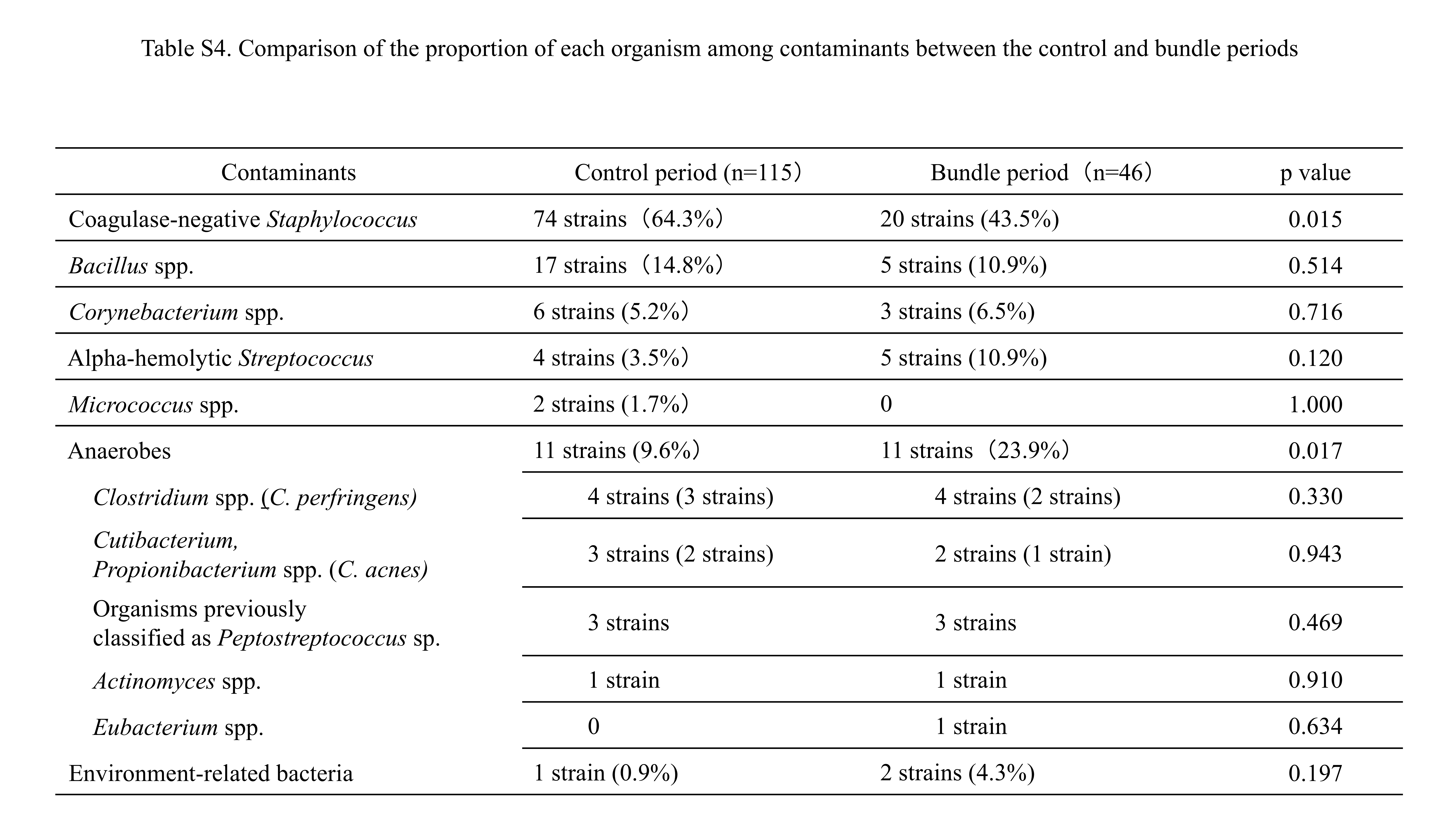

Supplement: S4 Table — (TIF) [file pone.0314649.s004.tif]

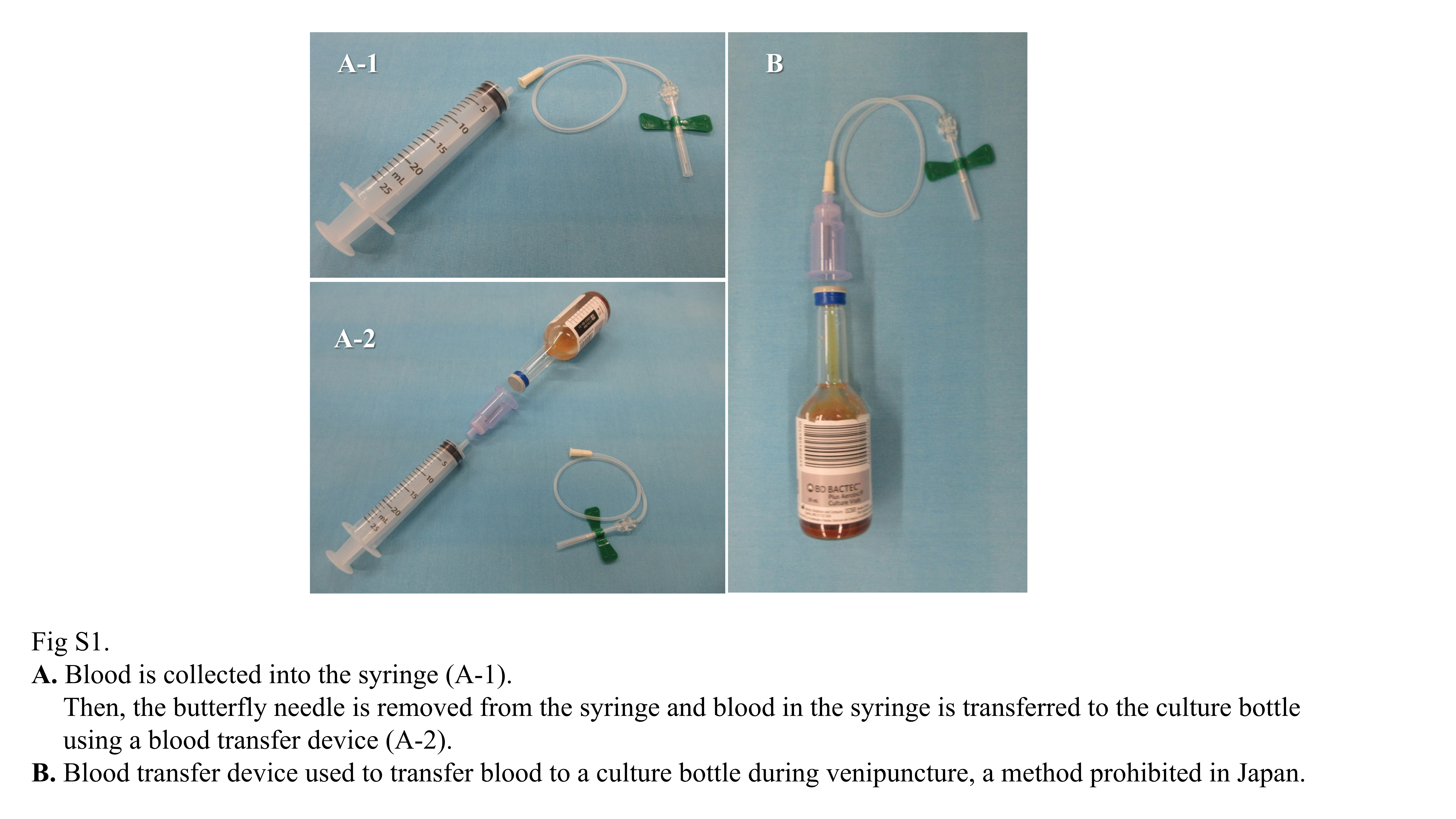

Supplement: S1 Fig — (A) Blood is collected into the syringe (A-1). Then, the butterfly needle is removed from the syringe and blood in the syringe is transferred to the culture bottle using a blood transfer device (A-2). (B) Blood transfer device used to transfer blood to a culture bottle during venipuncture, a method prohibited in Japan. (TIF) [file pone.0314649.s005.TIF]
