# Supplementary material for: Effect of a blood culture collection bundle on decreasing the contamination rate
Source: PLoS One. 2024 Dec 31;19(12):e0314649. doi: 10.1371/journal.pone.0314649 (PMC11687820; doi:10.1371/journal.pone.0314649)
Supplement: Attachment — Submitted filename: renamed_85bbb.docx [file pone.0314649.s007.docx]

Response to the reviewer comments

We wish to express our appreciation to the Reviewer for the insightful comments on our paper.
Reviewer #1: 
Major comments:
1. In the study, the authors discussed the effect of blood collection via intravascular lines on BC contamination only during the bundle period, but no data was provided in the control period. I am unsure how the authors concluded that bundle approach reduced the contamination, when there is no baseline or control group they have to compare with.

Response: Because blood collection through intravascular line is one of the increased risk factors for BC contamination, blood draw from line was evaluated in the control and the bundle period.

Line 288-293: Seventeen of 109 patients with BC contamination in the control period and 5 of 40 patients with BC contamination in the bundle period had a central venous catheter when venipuncture was conducted for BC; blood was collected through the line in 0 of 17 patients with contaminated BCs during the control period and in 1 of 5 patients with contaminated BCs during the bundle period. During both periods, no blood was drawn through an arterial line in any patients with BC contamination.

2. The study showed a seasonal variation in contamination rates, particularly for Bacillus species, which was reduced in bundle approach. This suggests that the overall contamination rate might be influenced by external factors, like environmental factors, seasonal changes beyond the intervention. The authors have not mentioned whether seasonality has any influences on any specific types of bacteria. This raises the question of whether the interventions' effectiveness might vary across seasons. Further investigation is needed to understand this relationship.

Thank you for the valuable comment to improve the quality of the paper. According to the reviewer comment, Table 5. (Comparison of the contaminant isolation rate between the control and bundle periods in August and September and in the remaining 10 months.) was added.

Line 325-329: The effectiveness of the bundle intervention was demonstrated not only in these 2 months but also in the other months, and there was a significantly lower BC contamination rate in the bundle period compared with the rate in the control period during the 10 months from October to July (0.93% vs. 1.64 %, p=0.007) (Table 5).

3. In this study institutional use of chlorhexidine-alcohol and sterile blood transfer devices not evaluated. Additionally, the study also did not assess their direct impact on BC contamination rates. This limit understanding of how consistently these materials were used. The authors should include this information for better understanding of the reader.

Response: We agree with the reviewer comments, and we demonstrated that key elements of the bundle to limit the contamination (The chlorhexidine-alcohol swab and the sterile blood transfer device) were not conducted in the control period

- The chlorhexidine-alcohol swab

Fig 1.

Line 240-243: In the control period, a povidone-iodine swab stick (Povidone Plus®, Kawamoto Corporation, Osaka, Japan) was exclusively used for skin preparation before BC blood collection. Chlorhexidine alcohol swabs were introduced for the first time in May 2022 for bundle implementation (Fig 1.).

- Sterile blood transfer devices

Line 267-275: Sterile blood transfer devices were introduced in December 2021 to transfer venous blood from a syringe to an evacuated blood collection tube for biochemical and peripheral blood cell examination. During the control period, use of the device was prohibited for transferring blood from a syringe to a BC bottle, and a conventional single-needle transfer technique was adopted. Use of the device to transfer blood to culture bottles was started following the introduction of the bundle, and institutional median monthly use significantly increased to 585 kits (IQR 424.5–646.5) in the bundle period (May 2022 to April 2024) comparing with that in the previous 6 months (p=0.008) (Fig 2).

Minor comments:
1. 1.Although the study mentioned about the potential sources of contamination, a detailed data should be provided on the frequency or types of contaminants identified. This information would be valuable in understanding the overall contamination picture.

Response: Detailed data was provided on the frequency or types of contaminants identified in Table 4. (Comparison of the blood culture contamination rate (per set) for each organism between the control and bundle periods)

2. The authors should include information on the specific characteristics of the study population (such as age, underlying conditions, or antibiotic use), as it would be beneficial for assessing the applicability of the findings to different patient groups.

Response: The comparison of the background in study group is needed. Table 1 was modified and median age, gender and No. of set obtained in emergency outpatient clinic (rate) was added. However underlying condition or antibiotic use in 2 study groups could not be evaluated. This was mentioned in the limitation of the study.

Line 415-418: Second, background information such as comorbidities or antibiotic use for patients in the two study groups was not collected. It would be beneficial for assessing the applicability of the findings to different patient groups.

Reviewer #2:

1. The English language and expression needs proper review from a native English speaker.

Response: Paper was corrected by a native English speaker

Line 431-433: Acknowledgment

We thank Analisa Avila, MPH, ELS, of Edanz (https://jp.edanz.com/ac) for editing a draft of this manuscript.

2. The materials and methods section shall be structured into sub sections with proper headings for each section

Response: Subsection with headings was prepared in the material and methods.

Study design; method of bacterial culture; definition of BC contamination; the bundle intervention for collecting blood samples for BC; adherence for the BC bundle; Evaluation for the BC contamination rate; and Statistical analyses

3. The figures, tables and figure legends be presented according to the journal's guidelines.

Base on the reviewer comment, the figures, tables and figure legends were corrected according to the journal’s guidelines
